# Pharmacologic Inhibition of Sphingomyelin Synthase (SMS) Activity Reduces Apolipoprotein-B Secretion from Hepatocytes and Attenuates Endotoxin-Mediated Macrophage Inflammation

**DOI:** 10.1371/journal.pone.0102641

**Published:** 2014-07-17

**Authors:** Bin Lou, Jibin Dong, Yali Li, Tingbo Ding, Tingting Bi, Yue Li, Xiaodong Deng, Deyong Ye, Xian-Cheng Jiang

**Affiliations:** 1 School of Pharmacy Fudan University, Shanghai, China; 2 Department of Cell Biology, SUNY Downstate Medical Center, Brooklyn, New York United States of America; Indian Institute of Science, India

## Abstract

Sphingomyelin synthase (SMS) plays an important role in plasma atherogenic lipoprotein metabolism, inflammation, and the development of atherosclerosis. To understand whether the impaired apoB secretion and inflammation response is a direct result from lack of SMS activity, in this study, we prepared a series of compounds that inhibit SMS activity. Further, we characterized Dy105, the most potent inhibitor. We found that Dy105 treatment significantly reduces SM levels in SM-rich microdomain on cell membranes. Moreover, we found that SMS inhibition reduces apoB secretion in a human hepatoma cell line and reduces the activation of NFκB and p38, a MAP kinase, in bone marrow derived macrophages. These studies provided further evidence that SMS activity regulates atherogenic lipoprotein metabolism and inflammatory responses. Pharmacologic inhibition of SMS may be a new therapy for atherosclerosis by reducing apoB secretion, and reducing inflammation.

## Introduction

In the developed countries, coronary heart disease (CHD) is the major cause of mortality. Now, statin therapy is the main option for CHD clinical management. Despite its potent efficacy, statin therapy is not always responsive and sometimes patient is intolerant to this therapy [1], [2], [3], [4], [5], [6]. Additional approaches are necessary to lower plasma atherogenic lipoprotein levels, and act synergistically with statins. Exploration of the sphingolipid metabolism is one of these approaches.

In human, serum or plasma sphingomyelin (SM) is considered as a risk factor for CHD [7], [8] and that SM levels are prognostic in patients with acute coronary syndrome [8]. Serum SM levels are enriched on atherogenic lipoproteins such as very low density lipoprotein (VLDL), low density lipoprotein (LDL), and chylomicron [9], [10]. The SM content of atherosclerotic lesions is elevated compared to normal arterial tissue [11]. Also subendothelial retention and aggregation of atherogenic lipoproteins play a very important role in atherogenesis [12], [13].

SM is produced by the transfer of phosphorylcholine from phosphatidylcholine to a ceramide in a reaction catalyzed by sphingomyelin synthases (SMS) [14]. SMS is the last enzyme for SM biosynthesis, Therefore, SMS activity should directly affect SM levels in cells and in the circulation.

SMS gene family consists of three members, SMS1, SMS2, and SMS related protein (SMSr). SMS1 expression is located in the trans-Golgi complex, while SMS2 is predominantly expressed in the plasma membranes [15], [16]. SMSr, the third member of the gene family, has no SMS activity but catalyses the synthesis of ceramide-phosphoethanolamine in the ER lumen [15], [17]. SMS1 and SMS2 are expressed in a variety of tissues and cells with different ratio. SMS1 is mainly expressed in macrophages [18], while SMS2 is mainly expressed in the liver [19]. It is reported that SMS1 and SMS2 expression is positively correlated with SM levels in cells and lipid rafts [20], [21], [22]. Our previous study also indicated that SMS1 and SMS2 expression in macrophages are positively related to the development of atherosclerosis [23], [24], and SMS2-mediated plasma SM reduction significantly decreases atherosclerosis in a mouse model [25]. Moreover, SMS2 deficiency decreased obesity and increases insulin sensitivity [26], [27], and SMS2 overexpression induced liver steatosis in mice [28]. These mouse studies suggested that SMS is a promising therapeutic target for CHD, despite many unresolved questions. Then SMS specific inhibitors could potentially worthy for basic scientific research and anti-atherosclerosis drug exploration.

In this study, we developed compounds that inhibit SMS activity and found that small compound-mediated SMS inhibition reduces cell plasma membrane SM levels, thus reducing hepatocyte apoB-containing lipoprotein (an atherogenic lipoprotein) secretion and reducing macrophage endotoxin-mediated inflammation. These studies indicated that inhibition of SMS by specific small compounds might be a promising approach in preventing the development of CHD.

## Materials and Methods

### Reagents

Dulbecco’s modified Eagle’s medium (DMEM) and fetal bovine serum (FBS) were from Thermo Scientific HyClone, Shiyi Biotechnology, Shanghai, China. L-[^35^S]methionine (specific activity >1175 Ci/mmol) were purchased from Perkin Elmer, Boston, MA. Chromatographically purified LPS from S. minnesota was from Sigma-Aldrich, St. Louis, MO. Dy105 and analogues was synthesized by Department of Medicinal Chemistry, School of Pharmacy in Fudan University, Shanghai, China. Phospho-p38 MAPK (Thr180/Tyr182) (D3F9) XP Rabbit anti-body were from Cell Signal Technologies, Shanghai, China. NFκBp65 subunit antibody was from Epitomics, Burlingame, CA, USA. Male (C57BL/6J) mice aged 8 weeks were from Central of Animal experiment in School of Pharmacy in Fudan University, Shanghai, China. All experimental procedures were approved by the Institutional Review Board of Fudan University.

### MTT assay for evaluating cell viability

Cells were treated with different concentration of inhibitor, and then incubated with lysenin. Cell viability was determined by MTT assay [29].

### Dy105 treatment and SMS activity assay

The Huh7 cells and bone marrow-derived macrophages were treated with various concentration of Dy105, the cell homogenate containing 50 mM Tris-HCl pH 7.5, 1 mM EDTA, 5% sucrose and 1 mM PMSF was centrifuged at 300 g for 5 min at 4°C, SMS activity in the supernatant was determined as described [22]. Total protein (200 µg) of supernatant was used for SMS activity assay.

### Cell Culture

#### a) Huh-7 culture

Huh-7 (a human hepatoma cell line) cells [29] were cultured in high-glucose DMEM with 10% fetal bovine serum, 2 mM glutamine, 100 U/ml penicillin and 100 µg/ml streptomycin at 37°C, 5% CO_2_. Exponential-phase cells were collected for further experiments.

#### b) Bone marrow-derived macrophage (BMM) culture

BMMs were obtained from femurs of 8 weeks old male C57BL/6J mice as described [32]. Cells were cultured in 100 mm Petri dishes with 10 ml of DMEM supplemented with 10% FBS and 20% L-cell-conditioned medium, as a source of macrophage colony-stimulating factor (M-CSF). After 6 days of culture, a homogeneous population of adherent identified as macrophages was obtained.

### Protein assay

Protein concentration was measured using a Bicinchoninic acid protein assay kit, (Pierce, Rockford, IL, USA) with bovine serum albumin as a standard.

### Lysenin-mediated cell mortality measurement

Lysenin-mediated cell mortality was measured as described by us before [30]. Briefly, after treated with various concentration of Dy105 for 17 hours, cells (Huh7 cells or BMM) were washed with PBS and 200 ng/ml of lysenin in serum-free was added in cells and incubated for another 2 hours at 37°C. MTT was used to determine cell viability [29].

### ApoB secretion in hepatocyte

The method has been described previously [31]. Briefly, Huh-7 cells were incubated for 17 hours with or without Dy105. The cells were then pulsed for 2 hours with 10 µCi of [^35^S]methionine in methionine-free DMEM with or without Dy105. The cells were washed twice with PBS and then chased in DMEM for 4 hours. After chase, the culture medium was collected and apoB from medium was precipitated by an anti-human apoB antibody (Abcam) together with protein A/G beads. [^35^S]apoB was separated by SDS-polyacrylamide gel electrophoresis (SDS-PAGE). The band corresponding to [^35^S]apoB was identified by an autoradiograph.

### Triglyceride-rich lipoprotein production rate measurement (in vivo)

Mice were injected with Poloxamer 407 to block the clearance of VLDL from the circulation. Plasma (150 µl) was collected at 0, 3, 6, 24 hours after injection. Plasma triglyceride and sphingomyelin levels were measured.

### Nuclear protein preparation and NFκB measurement

The method has been described before [30]. BMM were incubated with various concentration of Dy105 for 17 hours. The cells were collected, washed in cold PBS and lysed in buffer (10 mM HEPES, 10 mM KCl, 0.4% NP, 0.1 mM EDTA, 5 µg/ml aprotonin, 5 µg/ml leupeptin, 1 mM PMSF, pH 7.9). Nuclei were pelleted by centrifugation at 10000 rpm for 10 minutes at 4°C and then re-suspended in a buffer (20 mM HEPES, 400 mM NaCl, 1 mM EDTA, 10% Glycerol, 5 µg/ml aprotonin, 5 µg/ml leupeptin, 1 mM PMSF, 1 mM DTT, pH 7.9) and incubated for 30 minutes on ice, while continuously shock. The nuclear extract was recovered after centrifugation at 12000 rpm for 10 minutes at 4°C. Proteins were detected by Western blot and specific antibodies to NFκB-p65 as a probe. Histone 3 was used as a loading control.

### p38 MAPK activation analysis

BMM were incubated with various concentration of Dy105 for 17 hours before LPS treatment. The cells were collected and total proteins were extracted by RIPA Lysis Buffer supplemented with 1 mM PMSF, 10 µg/ml aprotonin, 10 µg/ml leupeptin, 1 mM DTT, pH 7.4. Protein samples were detected by Western blot and probed with the following primary antibodies: rabbit anti-human phosphor-p38 and β-actin antibody (Santa Cruz, CA, USA), followed by HRP-conjugated secondary antibody (Santa Cruz, CA, USA) and developed with ECL reagent (Beyotime Institute of Biotechnology, Shanghai).

### Statistical Analysis

Data are typically expressed as mean ± SD. Data between two groups were analyzed by the unpaired, two-tailed Student’s *t* test, and among multiple groups by ANOVA followed by the Student-Newman-Keuls (SNK) test.

## Results

### SMS activity inhibitors

**Figure 1:**
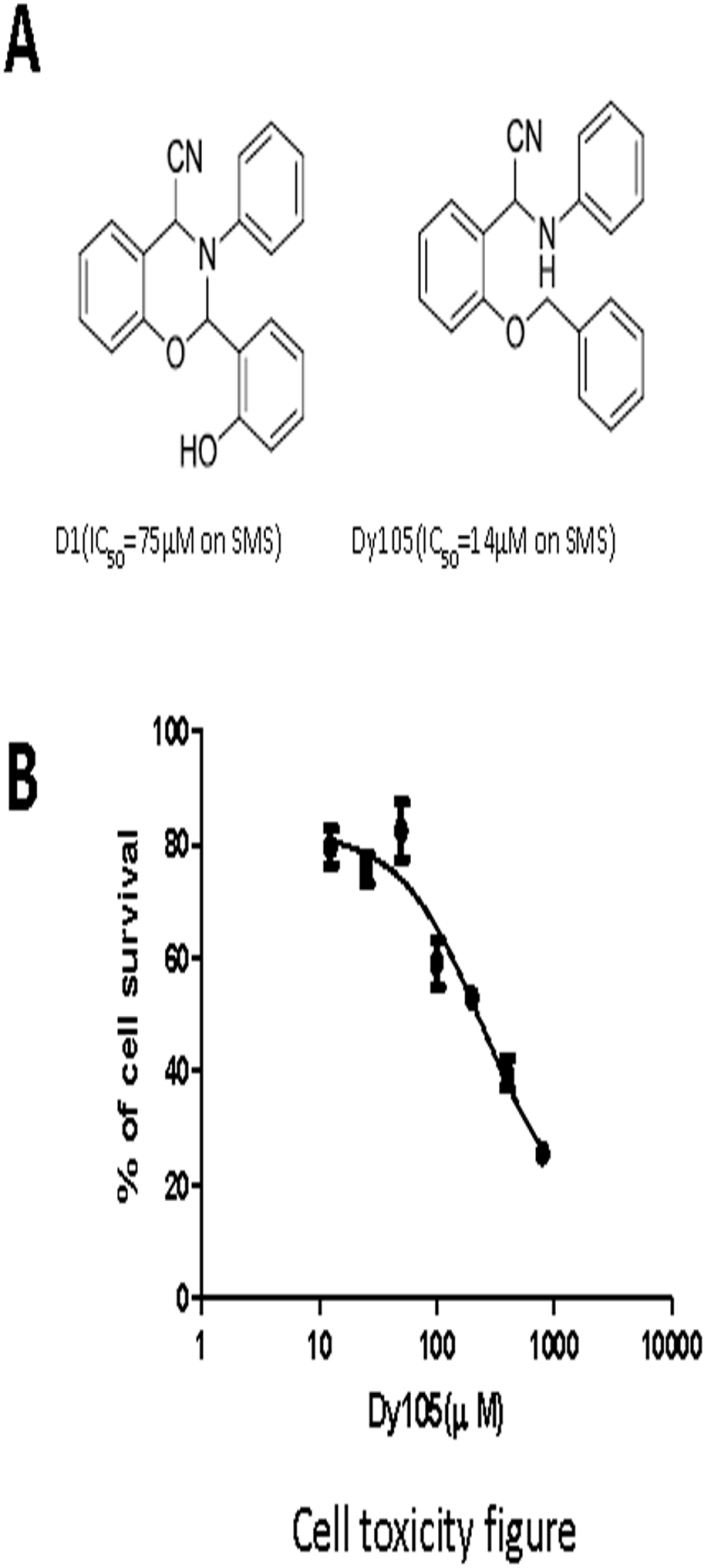
SMS inhibitors. Chemical structures of two SMS inhibitors were selected from structure-based virtual screening outcomes and were verified in biological assay. SMS activity was analyzed as reported before (Deng et al., 2012) and IC50 was calculated based on the analysis (panel A). Huh-7 cells were treated with Dy105 for 24 hours (panel B). Toxicity was assessed by MTT assay as described in “Materials and methods”. Values are mean ± SD, n = 4, *p<0.05.

Based on homology modeling and molecular dynamics simulation, we have reported a structural model of human SMS1 [32]. We generated a series of small compounds with SMS inhibiting activity. Among them, 2-(2-(benzyloxy)phenyl)-2-(phenylamino) acetonitrile (Dy105) is the most potent one with IC50 lower than 20 µM (Fig. 1A). Further, we found that Dy105 has mild cell toxicity and its CC50 is about 250 µM (Fig. 1B).

**Figure 2:**
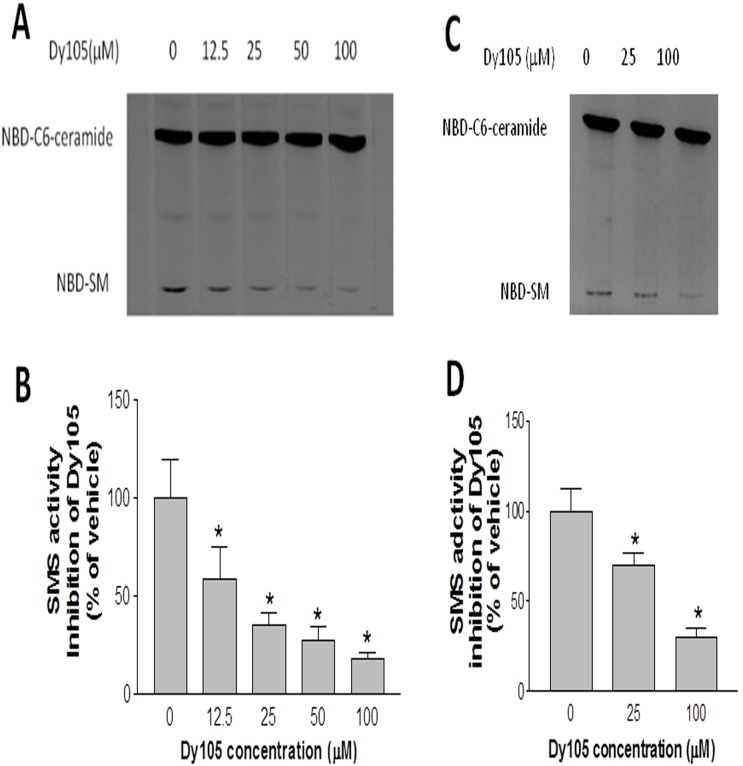
Dy105 inhibites SMS activity Huh-7 cells and bone marrow derived macrophages (BMM). SMS activity was measured in the presence of 0 to 100 µM Dy105 as described under “Materials and methods”. SMS activity in homogenate of Huh-7 cells (A) and quantitative display (B). SMS activity in BMM (C) and quantitative display (D). The data are expressed as percentage of control. Values are mean ± SD, n = 4, *p<0.05.

We next sought to evaluate the effect of Dy105 on Huh7 cells (a human hepatoma cell line) and bone marrow derived macrophages (BMM). We found that the compound significantly decreases Huh7 cell (Fig. 2A and B) and BMM (Fig. 2C and D) SMS activity in a dose dependent fashion.

**Figure 3:**
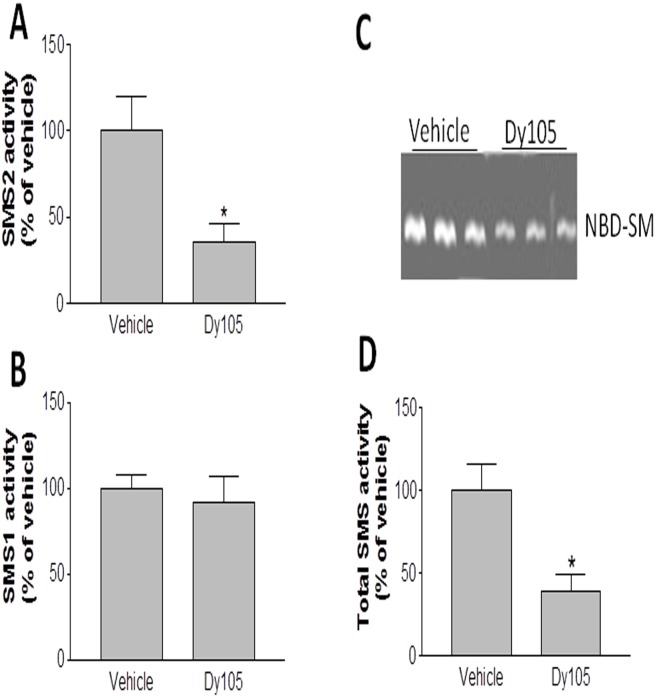
Dy105 inhibits both SMS1 and SMS2 activity. SMS activity was measured as described under “Materials and methods”. Homogenate of SMS1 KO mouse liver (200 µg of total protein) was used for Dy105-mediated SMS2 inhibition (A). Homogenate of SMS2 KO mouse liver (200 µg of total protein) was used for Dy105-mediated SMS1 inhibition (B). Wild type mice were injected (i.v.) with Dy105 (1.2 mg/kg), then the liver SMS activity was measured (C) and quantitation was displayed (D). Values are Mean ± SD., n = 4, *P<0.01.

We next sought to investigate whether Dy105 has SMS inhibitory specificity, we utilized SMS1 gene knockout (KO) mouse livers [24] to measure Dy105-mediated SMS2 inhibition, utilized SMS2 gene knockout (KO) mouse livers [30] to measure Dy105-mediated SMS1 inhibition, we found the compound can inhibit SMS2 but not SMS1 activity (Fig. 3A and B) in the liver. We will further evaluate this by using other tissues. So, Dy105 could have SMS2 inhibition specificity.

To further evaluate effect of Dy105 on SMS activity *in vivo*, we injected (i.v.) mice with Dy105 (1.2 mg/kg). Four hours later, the mice were sacrificed and livers were isolated. SMS activity in mouse liver homogenate was measured. We found that Dy105 significantly decreased liver SMS activity (Fig. 3C and D). As indicated by our previous study, Dy105 treatment significantly decreased plasma sphingomyelin and increased plasma ceramide levels [33]. Moreover, Dy105 treatment has no effect on both SMS1 and SMS2 mRNA levels (data not shown).

### SMS inhibition decreases SM levels on cell surface

**Figure 4:**
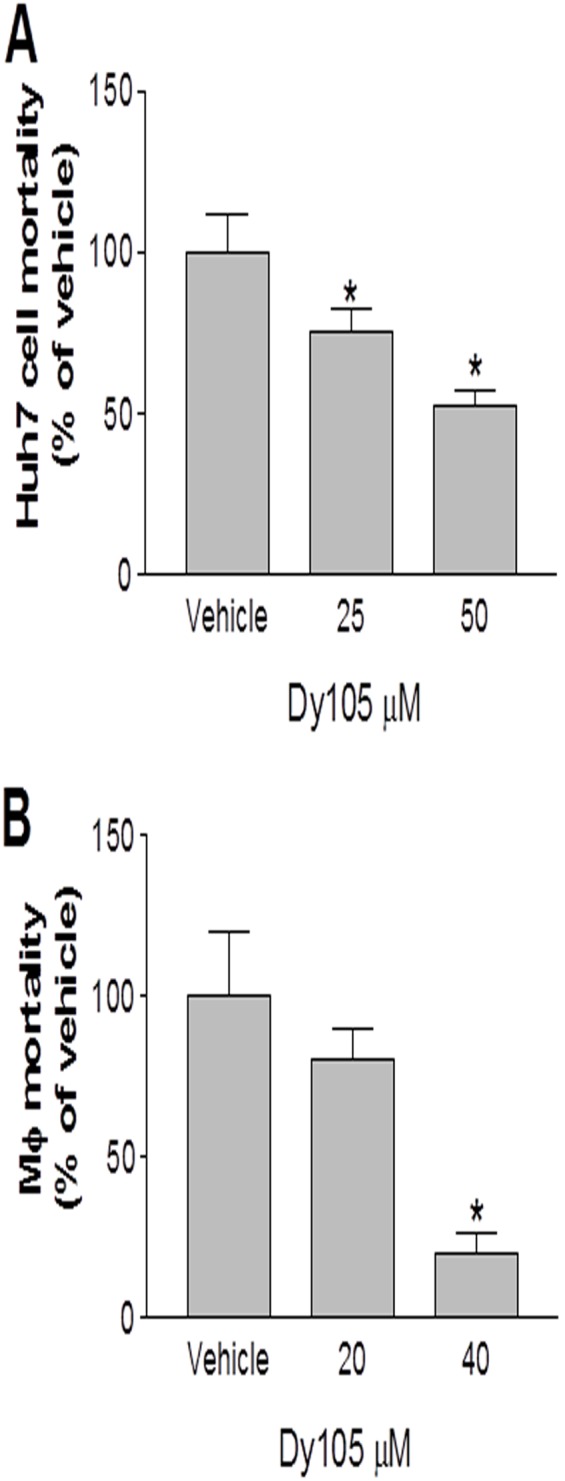
Dy105 treatment decreases lysenin-mediated cell mortality. Huh7 cells (A) and bone marrow derived macrophages (B) were treated with Dy105 for 17 h. Medium was removed. Lysenin (200 ng/ml) in PBS was added to the cells and cell mortality was monitored by MTT. Values are mean ± SD, n = 3, *p<0.05.

A major proportion of cellular SM is known to be present in the membrane rafts [22], [34]. We examine if inhibition of SMS by Dy105 impact on SM levels on plasma membrane. To estimate cell surface SM, we performed lysenin assay. Lysenin is a SM-binding protein, which specifically binds to SM-enriched microdomains in plasma membranes, inducing cell lysis. Therefore, lysenin-mediated cell mortality is associated with SM levels on cell surfaces. We treated Huh-7 cells and bone marrow derived macrophages with various concentration of Dy105 and then measured lysenin-mediated cytolysis. The cells treated with Dy105 showed significantly less sensitivity to lysenin-mediated cytolysis than control cells in a dose-dependent manner (Fig. 4A and B), indicating that Dy105 treatment significantly decreases SM levels in SM-rich microdomains on the cell surfaces.

### SMS inhibition decreases apoB-containing particle (BLp) secretion

**Figure 5:**
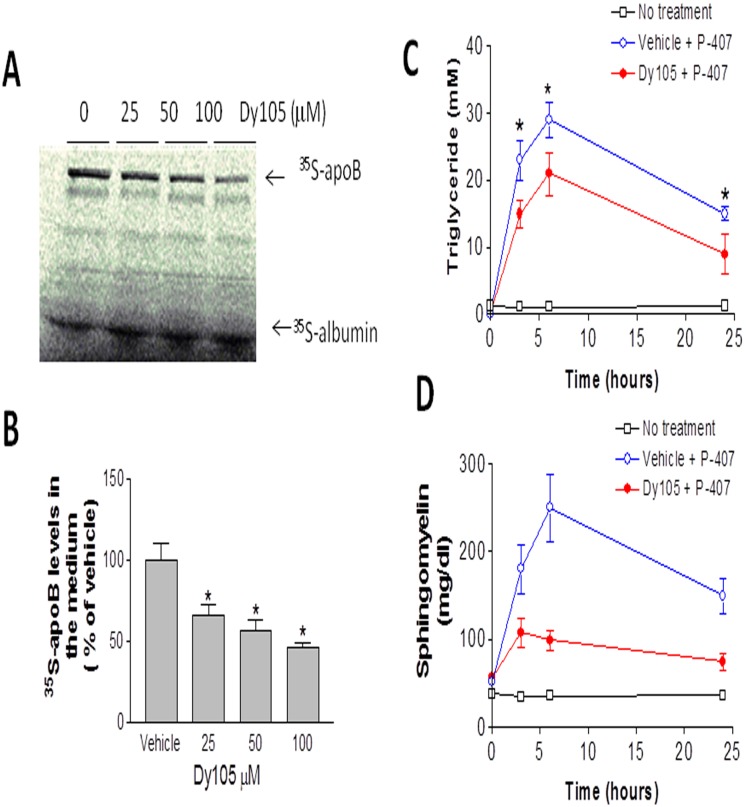
The effect of Dy105 on apoB-containing particle secretion. Huh7 cells were treated by Dy105 for 17[^35^S]methionine in the presence of vehicle or Dy105 for 2 hours. Medium were collected to detect [^35^S]apoB as described under “Materials and methods” (A) and the quantitation was displayed (B). Mice (wild type) were injected with Poloxamer 407 to block the clearance of VLDL from the circulation. Plasma (30 µl) was collected at 0, 3, 6, and 24 hours after injection. Plasma triglyceride (C) and sphingomyelin (D) levels were measured. Values are mean ± SD, n = 3, *p<0.05.

Apolipoprotein B (apoB) is the major protein component of VLDL and chylomicron (CM) [35], [36]. SM levels are enriched on VLDL and CM [9], [10]. In order to investigate whether SMS inhibition has an impact on apoB particle production, we treated Huh7 cells with Dy105 and then analyzed apoB secretion from the cells. We found that Dy105 treatment diminishes apoB secretion from Huh7 cells in dose-dependent manner (Fig. 5A and B), suggesting SMS inhibition in the liver could decrease atherogenic lipoprotein production.

To further investigate Dy105 effect, we examined the triglyceride-rich particle production rates *in vivo*. Wild type mice were injected with poloxamer 407 to block the clearance of VLDL from the circulation. We collected plasma 120 minutes after injection and measured triglyceride and sphingomyelin levels. We found that Dy105 treatment significantly decreases poloxamer 407-mediated triglyceride and sphingomyelin accumulation, suggesting that Dy105 has an effect on liver triglyceride/sphingomyelin-rich particle, i.e. VLDL, production (Fig. 5C and 5D).

### SMS inhibition attenuates endotoxin-mediated NFκB and MAP Kinase p38 activations

**Figure 6:**
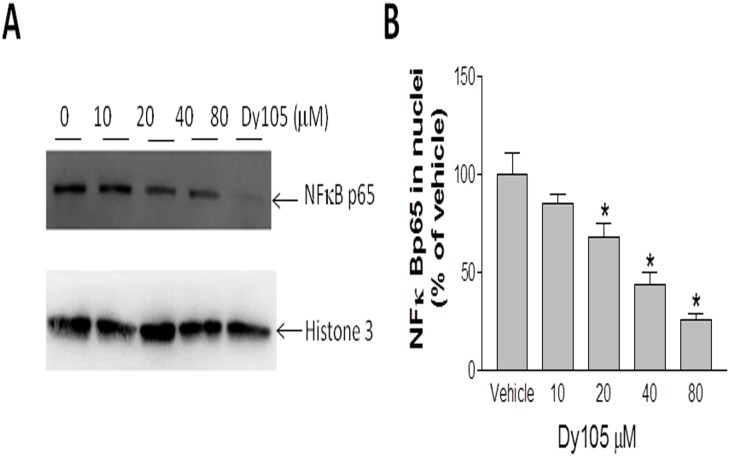
Western blot of nuclear NFκB. Bone marrow macrophages were treated by DY105 for 17-histone 3(H3) was used as loading controls (A). The quantitation was displayed (B). Values are mean ± SD, n = 3, *p<0.05.

Atherosclerosis is identified as inflammatory disease, due to macrophage-derived foam cells accumulated in the vessel wall and the production of chemokines, cytokines and growth factors [37]. Previously, we have reported that SMS1 or SMS2 deficiency in macrophages attenuate NFκB and MAP kinase activation, thus reducing the development of atherosclerosis in mouse models [39]. We attributed the mechanism to reducing SM levels in the lipid rafts [22]. To investigate whether Dy105 also have the same effect on macrophages, we treated bone marrow derived macrophages with different concentrations of Dy105 following LPS stimulation, and NFκB in nuclei was assayed. As shown in Figure 6A and B, Dy105 significantly decreased NFκB levels in nuclei in a dose-dependent manner.

**Figure 7:**
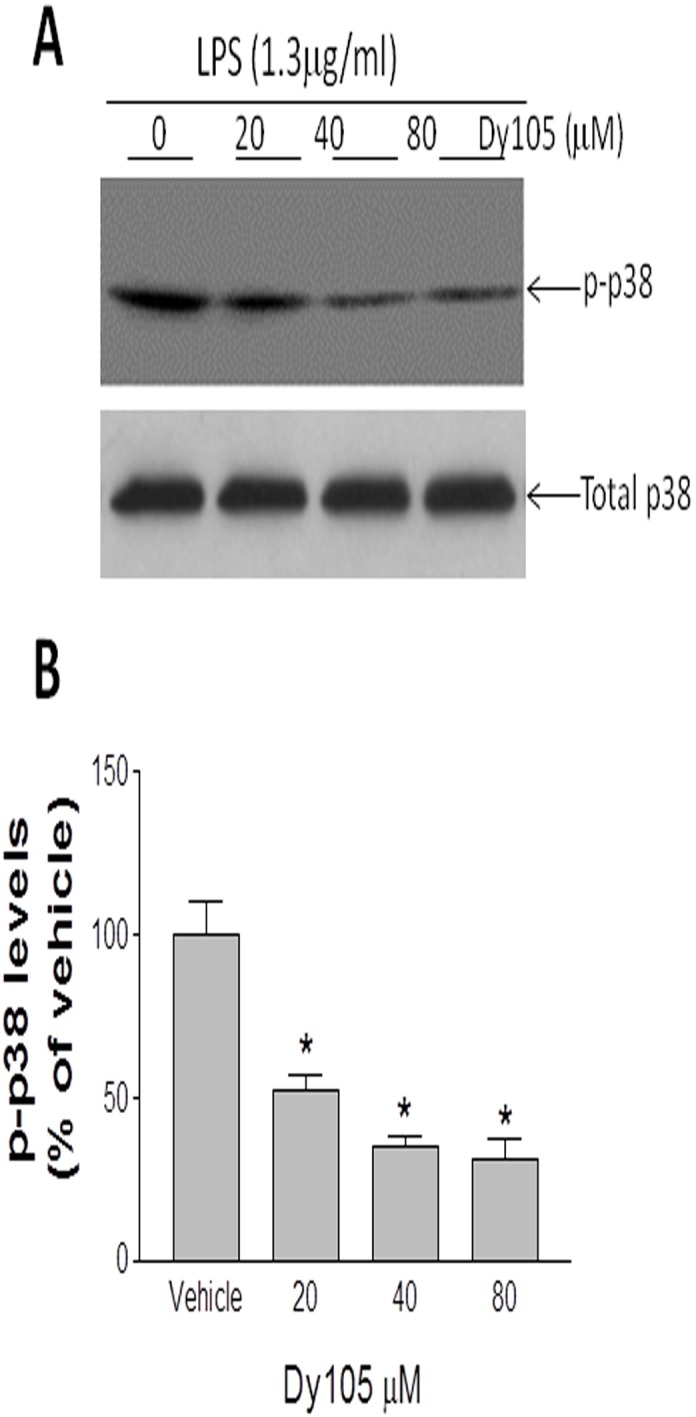
Effect of Dy105 on p38 activation. Bone marrow macrophages from C57BL/6J mouse were treated by Dy105 for 17 h following 30 min LPS stimulation. p38 phosphorylation were determined as described in “Materials and methods” (A). The quantitation was displayed (B). Values are mean ± SD, n = 3, *p<0.05.

It is conceivable that SMS inhibition should also influence signal pathways other than NFκB. To investigate this possibility, we did western blot for p38, a well known MAP kinase mediating inflammation (39), on Dy105-treated macrophages after LPS stimulation. We found that phospho-p38, the active form of the kinase, is decreased in the compound treated macrophages (Fig. 7A and B).

## Discussion

In this study, we have demonstrated that SMS inhibition by a small compound, Dy105, resulted into a significant decrease of plasma membrane SM levels, a significant reduction of BLp production from a hepatoma cell line, and a significant reduction of endotoxin-mediated NFκB and p38 activation in macrophages. These are useful tools to explore the impact of SM biosynthesis inhibition on the lipoprotein metabolism and the development of atherosclerosis.

SM is enriched on BLp [9], [10]. In atherosclerotic plaques SM-rich lipoproteins by enzymatic hydrolysis of sphingomyelin into ceramides induce aggregation [40], [41]. There are two options of preventing this atherogenic event. The first option is to reduce sphingomyelinase levels. However, human and mouse sphingmyelinase deficiency refers to a group of inherited severe metabolic disorders, Niemann–Pick disease [42], [43]. The second option is to reduce SM levels in the atherogenic lipoproteins by inhibiting SM biosynthesis, and then prevent atherogenicity. We have demonstrated in previous studies that an SMS deficiency causes lower plasma SM levels and then reducing atherosclerosis in a mouse model [25], [44]. As indicated in this study that compound Dy105, a SMS inhibitor, could also provide a very useful tool to explore the potential in the prevention of atherosclerosis.

The increase of hepatic apoB-containing lipoprotein production is considered as the principal defect in subjects with familial combined hyperlipidemia [38], [39], and is also considered as an important component of the dyslipidemia of diabetes and obesity [40], [41]. In this study, we showed that SMS inhibition decrease BLp secretion. There are two potential reasons for this observation, including that SM is an essential component involved in apoB lipidation or VLDL assembly and also BLp secretion is influenced by hepatocyte membrane SM levels [42].

SMS inhibition influence inflammatory responses via changing lipid-raft structure. One of the key regulators of inflammation, downstream of TLRs, is NFκB [43], [44], which has long been regarded as a pro-atherogenic factor, mainly because of its regulation of many pro-inflammatory genes (TNFα, IL-1, and IL-12) linked to atherosclerosis [45], [46], [47], [48] through different receptors [49], [50] in the macrophages. Many studies have also demonstrated a requirement for various MAP kinase signaling cascades in macrophage inflammatory responses. OxLDL-induced production of granulocyte macrophage colony stimulating factor (GM-CSF) in cultured macrophages is dependent on the activation of ERK and p38 activity [51]. OxLDL-induced foam cell formation is dependent on p38 activity [52]. Our previous studies indicated that SMS1 or SMS2 deficiency attenuates NFκB activation [23], [24]. In SMS2 knockdown cells, the recruitment of TNFR1 receptor to lipid rafts was blocked upon stimulation by TNFα [30]. These results suggesting the NFκB activity is potentially modulated by SMS2. Knockout of SMS2 in macrophages could diminish recruitment of TLR4-MD-2 complex to plasma membrane induced by LPS, and attenuate the activation of MAP kinases signal transduction pathways [30]. Taken together, these findings strongly suggest the critical role of SM, synthesized by SMS, in the normal function of plasma membrane TNFR1 and TLR4 receptors following stimulation by their respective ligands, such endotoxin and TNFα. SMS specific inhibitors may provide a useful tool for further investigation in this aspect.

In conclusion, SMS physiologically contributes to de novo SM biosynthesis and plasma membrane SM levels. A potent SMS inhibitor potentially affects the balance of SM, resulted into less apoB particle production and blunted NFκB and p38 responses to inflammatory/immunologic stimuli. Thus, inhibition of SMS activity may have an important impact on anti-atherogenic processes.
